# Apoptosis associated with Wnt/β-catenin pathway leads to steroid-induced avascular necrosis of femoral head

**DOI:** 10.1186/s12891-015-0606-2

**Published:** 2015-06-03

**Authors:** Chen Zhang, Yu-long Zou, Jun Ma, Xiao-qian Dang, Kun-zheng Wang

**Affiliations:** The First Department of Orthopaedics, the Second Affiliated Hospital of Medical College, Xi’an Jiaotong University, No.157 Xiwu Road, Xi’an, Shaanxi Province 710004 P. R. China; Department of Orthopaedics, Chongqing Medical University, Chongqing, 400010 P. R. China

**Keywords:** Steroid-induced avascular necrosis of femoral head, Apoptosis, β-catenin, c-Myc, Rat

## Abstract

**Background:**

The objective of the current study was to establish a rat model to investigate apoptosis in steroid-induced femoral head osteonecrosis occurring via the Wnt/β-catenin pathway.

**Methods:**

Male Sprague–Dawley (SD) rats were randomly divided into a control group (group A), model group (group B) and sFRP1 group (group C), each consisting of 24 rats, and the rats were intravenously injected with LPS (10 μg/kg body weight). After 24 h, three injections of MPS (20 mg/kg body weight) were administered intramuscularly at 24-h intervals. The rats in group C were injected intramuscularly with 1 μg/kg sFRP1 protein per day for 30 days, beginning at the time of the first MPS administration. The group A rats were fed and housed under identical conditions but received saline injection. All animals were sacrificed at weeks 2, 4 and 8 from the first MPS injection. Histopathological staining was preformed to evaluated osteonecrosis. Apoptosis was detected via quantitative terminal deoxynucleotidyl transferase (TdT) deoxyuridine triphosphate nick-end labelling (TUNEL) staining, caspase-3 activity assay, and detection of Bcl-2 and Bax protein expression by immunohistochemistry and Western blotting. Wnt/β-catenin pathway signalling molecules, including activated β-catenin and c-Myc, were detected by immunohistochemistry and Western blotting.

**Results:**

Typical osteonecrosis was observed in groups B and C. Apoptosis gradually increased with increasing time in both groups B and C. More severe osteonecrosis and apoptosis were observed in group C compared with group B. The expression levels of caspase-3 and Bax were higher while that of Bcl-2 was lower in group C compared with group B. The expression levels of activated β-catenin and c-Myc gradually decreased with increasing time in both groups B and C, and they were lower in group C compared with group B.

**Conclusions:**

The Wnt/β-catenin pathway is involved in the pathogenesis of early stage SANFH, as we have demonstrated in an SANFH rat model, and it may act through the regulation of c-Myc, which affects the cell cycle and cell apoptosis.

**Electronic supplementary material:**

The online version of this article (doi:10.1186/s12891-015-0606-2) contains supplementary material, which is available to authorized users.

## Background

Many studies have shown that glucocorticoid (GC) therapy is the most common non-traumatic cause of osteonecrosis of the femoral head, termed steroid-induced avascular necrosis of the femoral head (SANFH). The underlying mechanisms of SANFH are still unclear. Several mechanisms have been postulated, including coagulation, intraosseous hypertension, intravascular fat embolism, and compression of vessels by progressive accumulation of marrow fat stores.

Studies have demonstrated the strong association of osteoblast and osteocyte apoptosis with SANFH [1–4]. Weinstein and his colleagues have studied osteocyte apoptosis in femoral head specimens of 14 patients with necrosis of the femoral head using TUNEL staining assay [5], showing the appearance of marked osteocyte apoptosis in specimens from patients with SANFH, while apoptosis was absent or rare in specimens from patients with necrosis of the femoral head caused by other factors. Many studies have shown that osteocyte apoptosis occurs in the tissue of bone with SANFH [6].

The Wnt signalling pathway plays a critical role in embryonic development and regulation of apoptosis. Evidence suggests that the Wnt/β-catenin pathway is involved in apoptosis by regulating target downstream genes, such as c-Myc and cyclin D1 [7, 8]. The Wnt signalling pathway is related to the regulation of bone formation [9, 10]. Feng-Sheng Wang has conducted *in vivo* and *in vitro* testing of rats, demonstrating that glucocorticoids mediate the pathogenesis of osteoporosis by enhancing the expression of sFRP1 in bone tissue, leading to the negative regulation of the Wnt/β-catenin pathway, osteocyte apoptosis and bone mass loss [11]. An important role of Wnt/β-catenin in cell fate determination has been demonstrated for bone marrow mesenchymal stem cell (BMSC) differentiation [12–16]. Studies have indicated that the Wnt/β-catenin pathway may be associated with the pathogenesis of SANFH. However, few studies have been conducted to investigate the role of this pathway in SANFH.

In this study, we employed an animal model with SANFH induced by bacterial lipopolysaccharides combined with methylprednisolone to investigate apoptosis in SANFH occurring via the Wnt/β-catenin pathway. Apoptosis in the femoral head was evaluated after the animals were administered sFRP1, which is a Wnt/β-catenin pathway blocker. This study provides important information on the pathogenesis of SANFH that may facilitate the development of a novel treatment for this condition.

## Methods

### Animals

This study was performed in accordance with the National Institutes of Health guidelines for the use of experimental animals, and all animal protocols were approved by the Institutional Animal Care and Use Committee of Xi’an Jiaotong University. All surgeries were performed under sodium pentobarbital anaesthesia, and all efforts were made to minimize suffering. Male Sprague–Dawley (SD) rats (license number: SCXK [Shaanxi] 2008–008; weight 250–300 g; age: 12 months; SPF class) were obtained from the experimental animal centre of Xi’an Jiao Tong University. The rats were bred and maintained under a 12:12-h light–dark cycle with free access to food and water. The room temperature was set at 18 °C-25 °C, and the relative humidity was set at 40 %-60 %.

### Experimental protocols

The rats were weighed after feeding for 1 week. The early stage SANFH model was induced using a combination of lipopolysaccharides (LPS) and methylprednisolone (MPS). Seventy-two male SD rats were intravenously injected with LPS (10 μg/kg body weight). After 24 h, three injections of MPS (20 mg/kg body weight) were administered intramuscularly at 24-h intervals. To prevent infection, each rat was intraperitoneally injected with 100,000 U of penicillin. The rats were randomly divided into a control group (group A), model group (group B) and sFRP1 group (group C), each consisting of 24 rats. The rats in group C were injected intramuscularly with 1 μg/kg sFRP1 protein per day for 30 days, beginning at the time of the first MPS administration. The control group (group A) was fed and housed under identical conditions but received saline injection.

The rats in all groups were sacrificed by overdose of anaesthesia at weeks 2, 4 and 8 from the first MPS injection, and the femoral heads were harvested. The left femoral heads of all rats were preserved in a −70 °C cryogenic freezer immediately after sacrifice, and proteins were isolated for Western blot analysis. The right femoral heads were collected and immediately fixed with 10 % formalin (0.1 M phosphate buffer, pH 7.4) at 4 °C for 24 h.

### Histopathological staining

After the femoral heads harvested from the animals were fixed with 10 % formalin, they were washed with 0.2 M phosphate buffer (pH 7.4). Then, they were decalcified with 10 % EDTA and neutralized with sodium sulphate buffer for approximately 4 weeks. After decalcification, the tissues were embedded in paraffin and cut in the coronal plane into 4-μm thick sections with a microtome. Some of the sections were processed for routine haematoxylin-eosin staining to assess the general architecture and damage of the tissue. The stained sections were viewed at a magnification of 200 × and photographed with an Eclipse 50i optical microscope imaging system (Nikon, Co. Ltd., Toyko, Japan), and the images were analysed using Image-Pro-plus software (Media Cybernetics, Baltimore, MD).

### TUNEL staining

TUNEL staining was performed on paraffin sections using an *in situ* cell death detection kit (Jiancheng Bioengineering Institute, Nanjing, China) according to the manufacturer’s instructions. Sections were counterstained with haematoxylin. Quantitation was carried out by counting the number of TUNEL-positive cells in five randomly chosen fields of view on each slide at a magnification of 200x, and the values were averaged. Apoptosis was evaluated by determining the ratio of the number of TUNEL-positive cells to that of total cells.

### Caspase-3 activity assay

The activity of caspase-3 was detected with a Caspase-3 Colorimetric Assay Kit (Nanjing KeyGEN Biotech. Co., Ltd., Nanjing, China) according to the manufacturer’s protocol. Proteins from rat femoral head tissues were isolated with RIPA lysis buffer, and protein concentrations were determined with a BCA protein quantitation kit (Pierce, Rockford, IL, USA). Caspase-3 activity was expressed in terms of absorbance units (OD 405 nm).

### Western blot analysis

The protein levels of Bcl-2, Bax, activated β-catenin and c-Myc were detected by Western blotting. The specimens were washed twice in ice-cold PBS and subsequently lysed in RIPA buffer (990 μL RIPA and 10 μL phenylmethylsulphonyl fluoride), followed by grinding in liquid nitrogen for approximately 25 to 30 min. Supernatants were extracted into a centrifuge tube, and insoluble material was removed by microcentrifugation at 12000 r/min for 10 min in 4 °C. Proteins in the lysates were subsequently dissolved in SDS sample buffer (62.5 mM Tris/HCl, pH 6.8, 2 % w/v SDS, 10 % glycerol, 50 mM dithiothreitol, and 0.01 % w/v bromophenol blue), electrophoresed on SDS-PAGE with Tris-glycine running buffer, and electrophoretically transferred onto polyvinylidene difluoride membranes using a semi-dry apparatus (Bio-Rad, Hercules, CA, USA). The membranes were blocked with TBS/Tween20 (0.05 mM Tris, 0.15 mM NaCl, pH 7.6; 1 % Tween 20) containing 5 % w/v non-fat dried milk for 1 h and were then incubated in TBS/Tween20 with 5 % w/v non-fat dried milk supplemented with different rabbit anti-rat monoclonal antibodies (1:1000; Santa Cruz Biotechnology, USA) overnight at 4 °C. After the membranes were washed with TBS/Tween20 three times, they were incubated in the dark with a diluted (1:5000) secondary polyclonal antibody (goat anti-rabbit conjugated with peroxidase) in TBS/Tween-20 containing 5 % w/v non-fat dried milk at room temperature for 2 h with gentle shaking. Horseradish peroxidase-conjugated anti-β-actin (Santa Cruz Biotechnology, USA) was used as an internal control. Positive antibody interactions were visualized using an ECL-plus kit (Thermo Fisher Scientific Inc., USA) with an enhanced chemiluminescence substrate for horseradish peroxidase. Luminescent signals were detected and recorded with a CCD camera in a dark room and transmitted to a controller unit. Relative expression was quantified using densitometry and Quantity One software (Bio-Rad; Richmond, CA, USA). The obtained values were normalized to reference bands of β-actin. Each test was repeated three times.

### Immunohistochemistry

The femoral head tissue sections obtained by the above process were processed immunohistochemically to detect the presence of Bcl-2, Bax, activated β-catenin and c-Myc using the avidin-biotin-peroxidase complex (ABC) method. After permeabilization in phosphate-buffered saline (PBS) with 0.3 % Triton-X 100 (pH 7.4) for 30 min and then incubation in 0.3 % H_2_O_2_ for 1 h to block endogenous peroxidases, the sections were incubated with primary rabbit anti-rat monoclonal antibodies (1:200; Santa Cruz Biotechnology, USA) in a solution consisting of 1 % bovine serum albumin and 0.05 % sodium azide in 0.1 M PBS for 24 h at 4 °C. After three washes with PBS, the specimens were exposed to biotinylated goat anti-rabbit IgG diluted 1:200 in PBS for 4 h at room temperature. Next, the peroxidase reaction was developed for 10 min in 0.05 M Tris buffer (pH 7.6) containing 0.02 % 3, 3-diaminobenzidine tetrahydrochloride and 0.006 % H_2_O_2_. The sections were photographed using an Eclipse 50i optical microscope imaging system, and images were analysed with Image-Pro-plus software.

Positive staining for signalling molecules in the sub-chondral areas of the femoral heads was visualized as brown puncta and bundles distributed in the bone marrow, periosteum and bony trabeculae. The positive brown staining intensity of each antibody, as described in the manufacturer’s protocol, were used as positive controls. Samples without primary antibodies were used as negative controls. The intensities of immunostaining for Bcl-2, Bax, activated β-catenin and c-Myc in groups A, B and C were quantitatively analysed. The images obtained were analysed at a magnification of 200 × by performing quantitative integrated optical density measurements. The area of each analysed tissue was approximately the same. We evaluated the integrated optical density (the sum of all pixel intensity or density values in a given region). Ten different regions in each tissue were randomly selected, and the numerical values obtained from the ten regions were averaged to represent the expression of the specified marker in a given tissue.

### Statistical analysis

Quantitative data are expressed as the mean ± SD. Data were analysed using SPSS 19.0 software (SPSS, Chicago, IL. USA). For multiple comparisons, the LSD t test and the Student-Newman-Keuls (SNK) test were used to analyse significant differences. A p-value of less than 0.05 was considered statistically significant.

## Results

### Histopathological staining of osteonecrosis

A positive diagnosis of osteonecrosis was made on the basis of the diffuse presence of empty lacunae or pyknotic nuclei of osteocytes in the bone trabeculae, accompanied by surrounding bone marrow cell necrosis or myelofibrosis [18]. The histopathological staining of group A showed bone trabeculae with normal structures and osteocytes with normal morphologies. There were no necrotic bone marrow adipose tissues or haematopoietic cells. No obvious osteonecrosis was observed in group A. Typical osteonecrosis of femoral heads was observed by the histopathological staining of group B, and it was aggravated with increasing time. There were large fat cells in the marrow cavity and empty lacunae in the bone trabeculae. The bone trabeculae were in disorder and some of them were fractured. The osteonecrosis in group C was more serious than that in group B. At 8 weeks after the intervention, severe osteonecrosis appeared in group C. No haematopoietic cells were observed. There were a large number of empty lacunae in the thinning bone trabeculae that were distorted and fractured. The rates of empty lacunae were 7.13 ± 1.46 %, 9.13 ± 1.96 %, and 9.87 ± 1.64 % in group A at 2, 4 and 8 weeks, respectively, and these rates were 11.14 ± 1.95 %, 12.71 ± 1.80 %, and 16.57 ± 2.51 %, in group B and 15.71 ± 3.25 %, 23.28 ± 2.87 %, and 30.28 ± 3.68 % in group C, respectively. The rate of empty lacunae in group B was significantly higher than that in group A at each time point, and it was significantly higher in group C compared with both groups A and B at each time point. All of the above results are shown in Fig. 2.

**Fig. 1:**
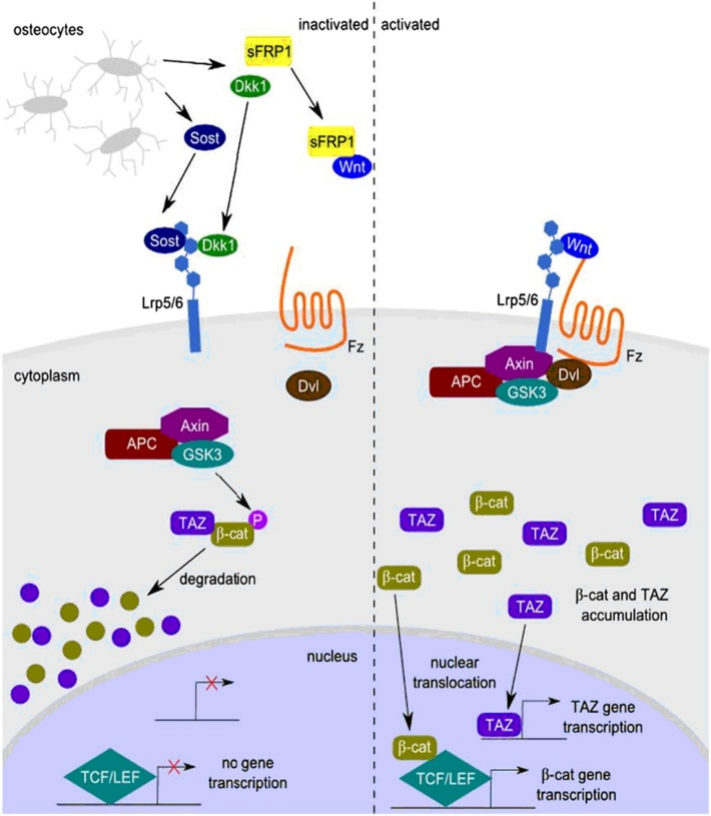
Schematic diagram of Wnt/β-catenin signalling pathway [17]

**Fig. 2:**
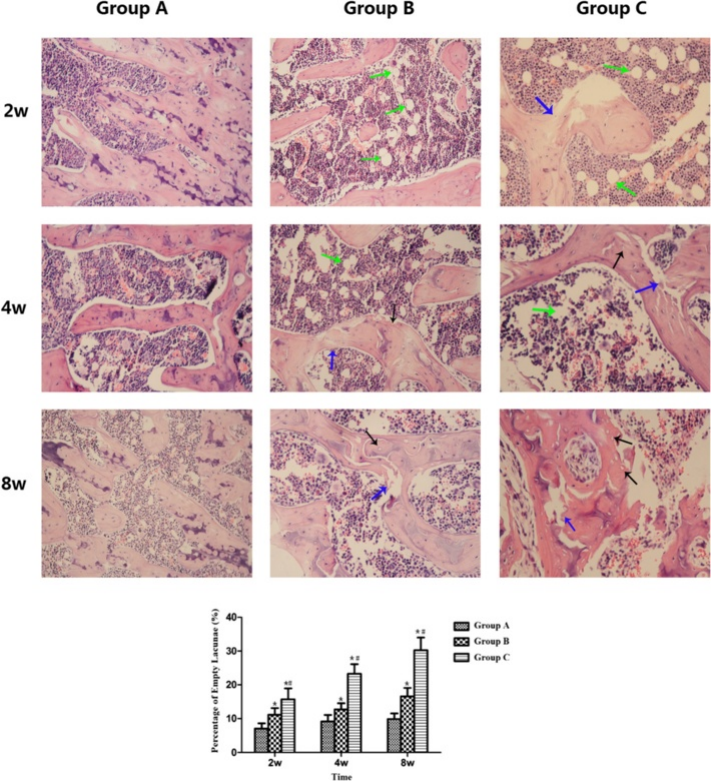
Visualization of femoral head osteonecrosis by haematoxylin-eosin staining. In group **a**, there was no visible necrosis of the bone or bone marrow. Typical osteonecrosis of the femoral heads was observed in groups **b** and **c**. At 8 weeks after the intervention, severe osteonecrosis appeared in group **c**. The rate of empty lacunae in group B was significantly higher than that in group A at each time point, and it was significantly higher in group **c** compared with both groups **a** and **b** at each time point. (* compared with group **a**, *P* < 0.05; and # compared with group **b**, *P* < 0.05. The blue arrow indicates the bone trabeculae; the green arrow shows the fat cells; and the black arrow shows the empty lacunae)

### TUNEL staining of apoptosis

**Fig. 3:**
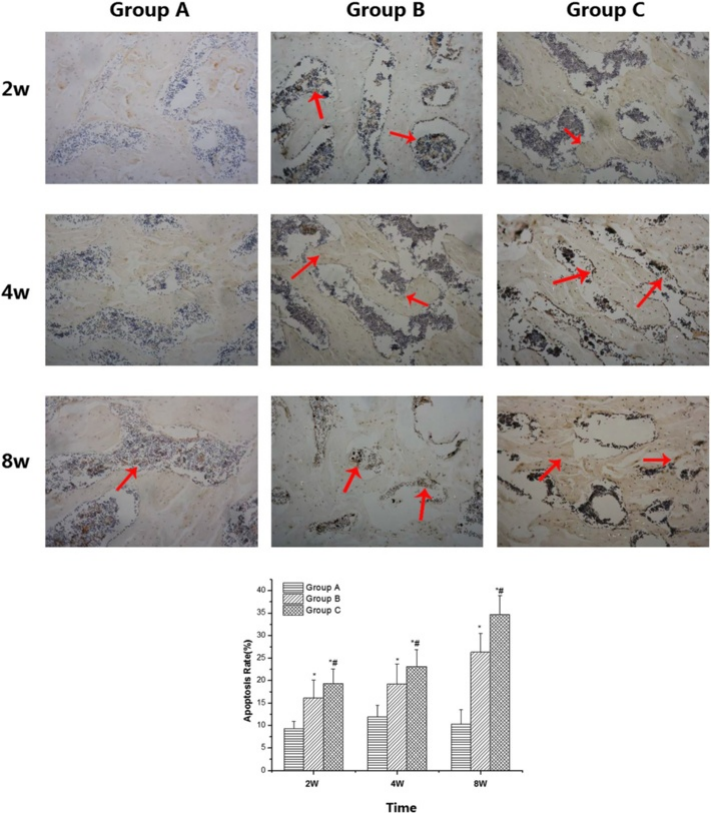
TUNEL staining of apoptosis. There was no abnormal apoptosis detected in group **a** at any of the time points. TUNEL staining showed gradual increases in apoptosis in both groups **b** and **c**, especially group **c**. The rate of apoptosis in group **b** was significantly higher than that in group **a** at each time point, and it was significantly higher in group **c** compared with both groups **a** and **b** at each time point. (* compared with group **a**, *p* < 0.05; and # compared with group **b**, *P* < 0.05. The red arrow shows the TUNEL-positive cells)

TUNEL assay was performed to detect apoptotic nuclei in bone tissue sections, and the results are shown in Fig. 3. There was no abnormal apoptosis in group A at any of the time points. TUNEL staining showed gradual increases in the rate of apoptosis in both groups B and C, especially group C. The rates of apoptosis were 9.31 ± 1.62 %, 11.91 ± 2.60 %, and 10.25 ± 3.32 % in group A at 2, 4 and 8 weeks, respectively, and they were 16.13 ± 3.98 %, 19.21 ± 4.37 %, and 26.32 ± 4.13 % in group B at 2, 4 and 8 weeks and 19.35 ± 3.02 %, 23.16 ± 3.77 %, and 34.75 ± 4.01 % in group C, respectively. The rate of apoptosis in group B was significantly higher than that in group A at each time point, and that in group C was significantly higher than those in both groups A and B at each time point.

### Caspase-3 activation, Bax and Bcl-2 protein expression

**Fig. 4:**
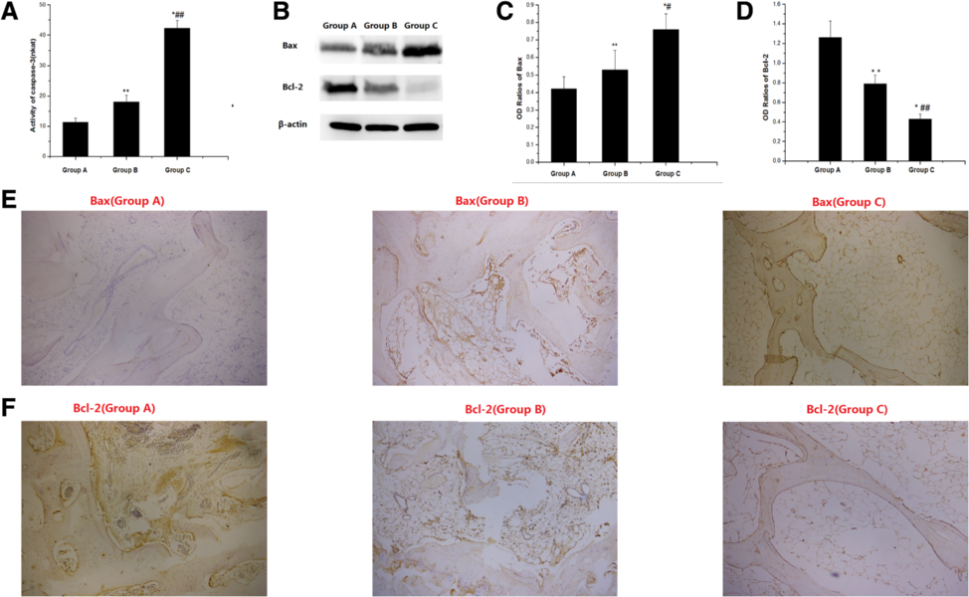
Caspase-3, Bax and Bcl-2 expression. **a**: Caspase-3 activity in the femoral head bone tissue. *p < 0.05 vs. group A; **p < 0.01 vs. group A; and ^##^ *p* < 0.01 vs. group B. **b**: Western blotting of Bax and Bcl-2. **c**: OD ratios for BAX. *p < 0.05 vs. group A; **p < 0.01 vs. group A; and ^#^ *p* < 0.05 vs. group B. **d**: OD ratios for Bcl-2. *p < 0.05 vs. group A; **p < 0.01 vs. group A; and ^##^ *p* < 0.01 vs. group B. **e**: Immunohistochemical staining of Bax. Positive staining for Bax was observed in groups B and C (magnification 200×). **f**: Immunohistochemical staining of Bcl-2. Positive staining for Bcl-2 in groups B and C was significantly weaker than that in group A (magnification 200×)

To detect apoptosis pathway signalling molecule expression, caspase-3 enzyme activity and Bcl-2 and Bax expression in the femoral head were determined at 8 weeks after the first MPS injection. Compared with group A, the activity of caspase-3 in group B increased significantly, and its activity in the femoral head in group C was significantly increased compared with that in group B (Fig. 4a).

Bax protein expression was significantly increased in group B compared with that in group A and it was obviously increased in group C compared with that in group B, according to the results of Western blot assay and immunohistochemical staining (Fig. 4b, c and e). Bcl-2 protein expression showed the opposite trend as Bax protein expression, exhibiting a significant decreases in groups B and C according to the results of Western blot assay and immunohistochemical staining (Fig. 4b, d and f).

### Protein levels of signalling molecules

**Fig. 5:**
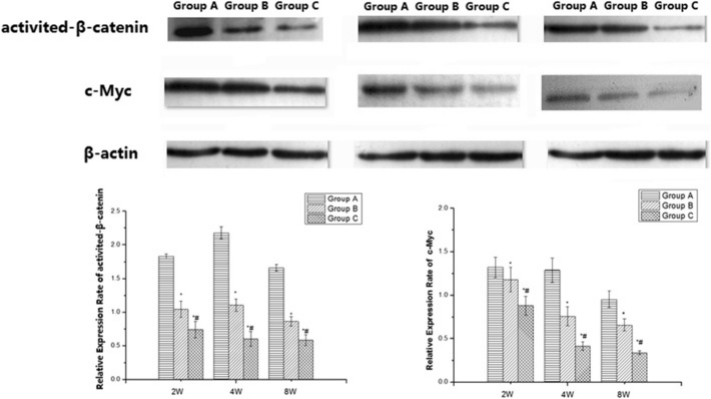
The protein levels of signalling molecules in the rat femoral heads of all groups. The protein expression levels of activated β-catenin and c-Myc did not obviously change with increasing time in group **a**, and they gradually decreased in both groups **b** and **c**, especially group **c**. Their levels were significantly lower in both groups **b** and **c** compared with those in group **a** at each time point, and they were also significantly lower in group **c** compared with group **b** at each time point. (*compared with group **a**. *P* < 0.05; and # compared with group **b**, *P* < 0.05)

To investigate the protein expression levels of activated β-catenin and c-Myc in the femoral head in each group, Western blot was conducted, and the results are shown in Fig. 5. The protein expression levels of activated β-catenin and c-Myc did not obviously change with increasing time in group A, and they gradually decreased with increasing time in both groups B and C, especially group C. The protein levels of activated β-catenin and c-Myc in both groups B and C were significantly lower than those in group A at each time point, and they were also significantly lower than those in group B at each time point.

### Immunohistochemistry of signalling molecules

**Fig. 6:**
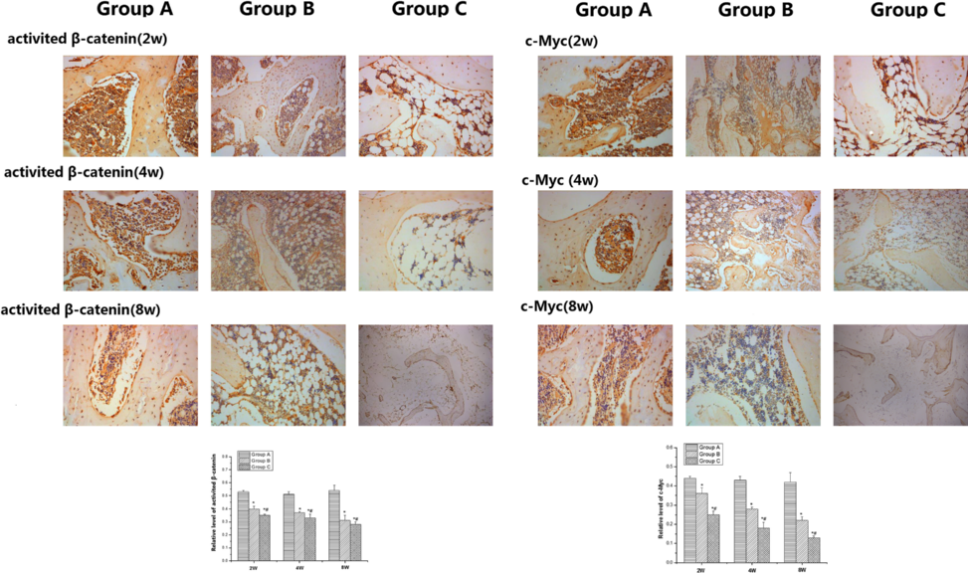
The positive staining of signalling molecules in the three groups by immunohistochemistry. The positive staining of the two proteins was observed in group **a**, and no significant differences were detected with increasing time. The positive staining intensities of activated β-catenin and c-Myc in both groups **b** and **c** were significant weaker than those in group **a**. Further, they gradually decreased with increasing time in both groups **b** and **c** and were significantly weaker in group **c** compared with those in group **b**. (* compared with group **a**, *P* < 0.05; and # compared with group **b**, *P* < 0.05)

Immunohistochemistry was employed to detect activated β-catenin and c-Myc in the femoral head in each group. The results are shown in Fig. 6. The positive staining of the two proteins was detected in group A, and there was no significant difference detected with increasing time. The positive staining intensities of activated β-catenin and c-Myc in both groups B and C were significantly weaker than those in group A. Further, these staining intensities gradually decreased with increasing time in both groups B and C, and they were significantly weaker in group C compared with those in group B.

## Discussion

Studies have shown that osteocyte and osteoblast apoptosis is associated with SANFH. Glucocorticoids can induce the apoptosis of cells, including osteocytes and osteoblasts. These findings may indicate an important mechanism leading to steroid-induced avascular necrosis of the femoral head [6]. In this study, TUNEL staining showed that the highest and lowest rates of apoptosis occurred in the sFRP1-treated group and control group, respectively. We have demonstrated that apoptosis takes place during the pathogenesis of SANFH.

Caspase-3 is an effector caspase that is involved in many forms of apoptosis [19]. It operates as a key effector enzyme in cell death through the receptor-mediated (Fas/FasL) or mitochondrial-dependent (Bax/Bcl-2) induction of apoptosis. In this study, caspase-3 activity was increased in the model animals, and with sFRP intervention, its expression was significantly increased. The dynamic balance of Bcl-2 and Bax plays a key role in promoting or inhibiting apoptosis [20]. The current study showed that glucose induced apoptosis in the animals, accompanied by an increase in Bax expression and a decrease in Bcl-2 expression, which were aggravated by sFRP treatment. These results indicate that apoptosis in steroid-induced femoral head osteonecrosis occurring via the Wnt/β-catenin pathway is associated with increases in Bax expression and caspase-3 activation and a decrease in Bcl-2 expression.

The Wnt pathway is very important for embryonic development, the regulation of tissues and apoptosis. Studies have demonstrated that target downstream genes of the Wnt/β-catenin pathway, such as c-myc and cyclin D1, are involved in apoptosis [7, 8]. Wnt protein signals were transmitted through interactions with Fzd family proteins and the low-density lipoprotein receptor-related protein (LRP5/6). One of the key regulators in the Wnt pathway is β-catenin. Cytoplasmic β-catenin can translocate into the nucleus and bind to T cell factor (TCF)/lymphoid enhancer-binding factor (LEF) to stimulate the transcription of target genes, including Bcl-2, c-myc and cyclin D1, which can inhibit apoptosis [8]. When the Wnt pathway is blocked, the rate of apoptosis may be increased. In this study, we conjecture that apoptosis was increased by the inhibition of the Wnt/β-catenin pathway, which was suppressed by glucocorticoids in the model group. Thus, the Wnt/β-catenin pathway is involved in the pathogenesis of SANFH.

Several secreted protein families antagonize Wnt/β-catenin signalling, including the secreted Frizzled-related proteins (sFRP), Wnt inhibitory factor (WIF) and the secreted Dickkopf (DKK) family proteins (DKK1-4). We chose sFRP1, which can be commercially purchased, to antagonize the Wnt/β-catenin signalling pathway. The sFRP protein belongs to a family of secreted glycoproteins located on chromosome 8p12-11.1 [21, 22]. SFRP receptor competitively or directly binds to the Wnt protein, thereby blocking the expression of Wnt in embryonic and adult tissues and organs. Previous studies examining the use of sFRP1 to inhibit the classical Wnt pathway have shown that the deletion of this protein has a substantial effect on the bone formation process. The inhibition of sFRP1 has been shown to promote bone formation *in vitro* [23–25]. High levels of bone mineral density and bone formation have been reported in sFRP1 knockout mice [26, 27], and sFRP1 knockout also leads to endochondral bone formation [28] and may even speed up fracture healing in animals [29]. In the current study, the expression levels of activated β-catenin and c-Myc gradually decreased with increasing time in both groups B and C, and they were lower in group C compared with group B. These findings indicate that abnormalities in the Wnt/β-catenin signalling pathway are involved in early stage SANFH.

One limitation of this study was that the longest observation period was 8 weeks after MPS injection. A prolonged observation period may better elucidate the pathogenesis of osteonecrosis. There are many subsequent investigations that need to be performed in future studies, including determinations of the mechanisms by which glucocorticoids affect the Wnt/β-catenin signalling pathway and the roles of this pathway and other pathways related to sFRP1 in other types of femoral head necrosis.

## Conclusions

In summary, we have demonstrated a role of apoptosis in the pathogenesis of SANFH. Glucocorticoids caused the decreased expression of activated β-catenin and c-Myc, which are downstream genes of the Wnt/β-catenin signalling pathway. When this pathway was antagonized by sFRP1, expression of activated β-catenin and c-Myc decreased dramatically, leading to an increase in apoptosis. Our findings indicate that abnormalities in the Wnt/β-catenin signalling pathway are involved in early stage SANFH. In addition, the underlying mechanism of this condition may be related to the regulation of c-Myc expression, leading to osteocyte and osteoblast apoptosis.
